# Human B Cell Responses to Dominant and Subdominant Antigens Induced by a Meningococcal Outer Membrane Vesicle Vaccine in a Phase I Trial

**DOI:** 10.1128/msphere.00674-21

**Published:** 2022-01-26

**Authors:** Christine S. Rollier, Christina Dold, Leanne Marsay, Aline Linder, Christopher A. Green, Manish Sadarangani, Gunnstein Norheim, Jeremy P. Derrick, Ian M. Feavers, Martin C. J. Maiden, Andrew J. Pollard

**Affiliations:** a Oxford Vaccine Group, Department of Paediatrics, University of Oxford and the NIHR Oxford Biomedical Research Centre, Oxford, United Kingdom; b Norwegian Institute of Public Health, Oslo, Norway; c School of Biological Sciences, Faculty of Biology, Medicine and Health, Manchester Academic Health Science Centre, The University of Manchester, Faculty of Life Sciences, University of Manchester, Manchester, United Kingdom; d National Institute for Biological Standards and Control, Hertfordshire, United Kingdom; e Department of Zoology, University of Oxford, Oxford, United Kingdom; University of Maryland School of Medicine

**Keywords:** *Neisseria*, bacteria, genetic modification, infection, meningitidis, meningitis, meningococcal, outer membrane, outer membrane proteins, outer membrane vesicles, vaccine, vesicles

## Abstract

Neisseria meningitidis outer membrane vesicle (OMV) vaccines are safe and provide strain-specific protection against invasive meningococcal disease (IMD) primarily by inducing serum bactericidal antibodies against the outer membrane proteins (OMP). To design broader coverage vaccines, knowledge of the immunogenicity of all the antigens contained in OMVs is needed. In a Phase I clinical trial, an investigational meningococcal OMV vaccine, MenPF1, made from a meningococcus genetically modified to constitutively express the iron-regulated FetA induced bactericidal responses to both the PorA and the FetA antigen present in the OMP. Using peripheral blood mononuclear cells collected from this trial, we analyzed the kinetics of and relationships between IgG, IgA, and IgM B cell responses against recombinant PorA and FetA, including (i) antibody-secreting cells, (ii) memory B cells, and (iii) functional antibody responses (opsonophagocytic and bactericidal activities). Following MenPF1vaccination, PorA-specific IgG secreting cell responses were detected in up to 77% of participants and FetA-specific responses in up to 36%. Memory B cell responses to the vaccine were low or absent and mainly detected in participants who had evidence of preexisting immunity (*P* = 0.0069). Similarly, FetA-specific antibody titers and bactericidal activity increased in participants with preexisting immunity and is consistent with the idea that immune responses are elicited to minor antigens during asymptomatic *Neisseria* carriage, which can be boosted by OMV vaccines.

**IMPORTANCE** Neisseria meningitidis outer membrane vesicles (OMV) are a component of the capsular group B meningococcal vaccine 4CMenB (Bexsero) and have been shown to induce 30% efficacy against gonococcal infection. They are composed of multiple antigens and are considered an interesting delivery platform for vaccines against several bacterial diseases. However, the protective antibody response after two or three doses of OMV-based meningococcal vaccines appears short-lived. We explored the B cell response induced to a dominant and a subdominant antigen in a meningococcal OMV vaccine in a clinical trial and showed that immune responses are elicited to minor antigens. However, memory B cell responses to the OMV were low or absent and mainly detected in participants who had evidence of preexisting immunity against the antigens. Failure to induce a strong B cell response may be linked with the low persistence of protective responses.

## INTRODUCTION

Neisseria meningitidis is a Gram-negative diplococcus, which is classified into serogroups according to the immunochemistry of the surface polysaccharide capsule with six capsular groups (corresponding to serogroups A, B, C, W, Y, and X) responsible for most invasive meningococcal disease (IMD) worldwide (1). Effective conjugate protein-polysaccharide vaccines are available for meningococci expressing capsular groups A, C, W, and Y. However, they have not been developed for capsular group B (MenB) because this polysaccharide is poorly immunogenic and is chemically identical to polysialyl decorations of the human neural cell adhesion molecule (NCAM) (2). Therefore, vaccines to target meningococci expressing group B capsule have been developed using subcapsular antigens either as recombinant proteins or outer membrane vesicles (OMVs) (3, 4).

OMVs are naturally produced by N. meningitidis in culture and contain multiple antigens (5, 6), including subcapsular antigens in their natural conformations, such as the immunodominant outer membrane protein (OMP) Porin A (PorA), which is a target for protective antibodies (7). OMV vaccines have been used to control outbreaks of MenB disease caused by a particular hyperinvasive meningococci expressing certain PorA variants (8). However, PorA proteins are highly variable, and OMV vaccines elicit mainly strain-specific protection, especially in children (7). A meningococcal OMV vaccine used in New Zealand (MeNZB) has been shown to induce partial protection against gonococcal infection, although the gonococcus does not express a PorA protein (9). Consequently, antibody responses elicited to antigens other than PorA (10) may have bactericidal activity against the gonococcus and, potentially, other meningococci (11).

Comparative analysis of the predicted surface proteins among 970 gonococcal genomes with the MeNZB proteome showed that 12 OMPs, including PorB, RmpM, PilQ, OpcA, FetA, Omp85 (BamA), and LbpA, were abundantly and consistently present in MeNZB. Their genes were present in the N. gonorrhea genomes. FetA is an integral outer membrane protein and a TonB-coupled iron transporter with an external subdomain, which is the target for antibody binding (12). The FetA sequence similarity was 89.5 to 100% in gonococci (13). The FetA protein was previously shown to induce bactericidal antibodies against meningococci (14). However, its immunogenicity in humans is poorly studied (15), and little is known about the immunogenicity of subdominant antigens contained in OMVs.

MenPF1 is an investigational OMV vaccine, produced from a meningococcus genetically modified to constitutively express the normally iron-regulated protein FetA. Safety, tolerability, and immunogenicity of this vaccine have been examined in healthy adults (16) and constitutive expression resulted in FetA (7.7% total protein) and PorA (21.8%) that simultaneously induced serum bactericidal responses. The clinical trial enabled an exploration of the kinetics and relationships between B cell responses against the PorA and FetA antigens. Here, we characterized the PorA and FetA-specific IgG, IgA, and IgM antibody-secreting cell and memory B cell responses to MenPF1 vaccination and functional activity as measured by the bactericidal and opsonophagocytic activities.

## RESULTS

### IgG-specific antibody-secreting cell responses are detected against both antigens.

**FIG 1:**
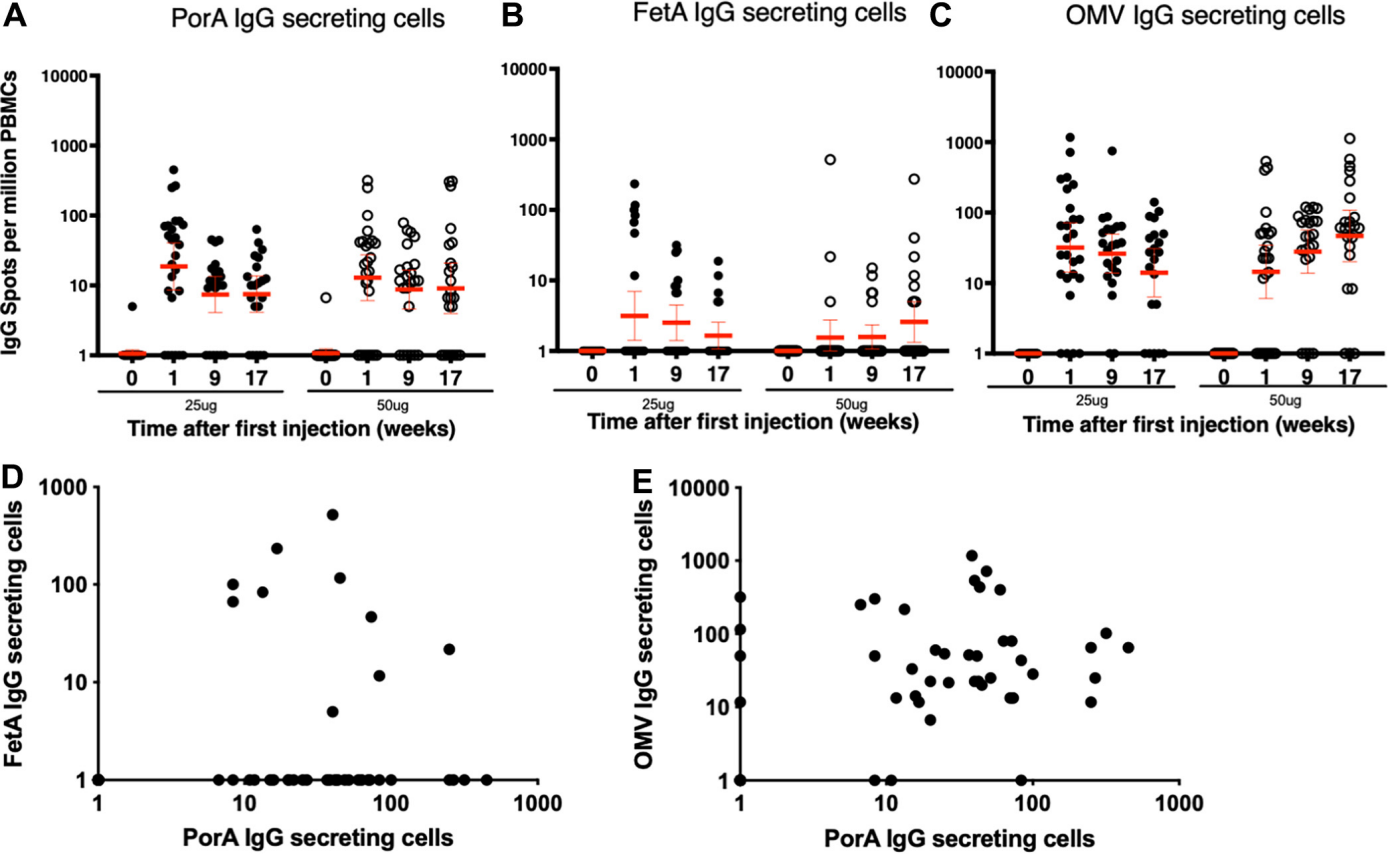
Kinetics of IgG-producing antibody-secreting cells (ASC) detected by *ex vivo* ELISPOT before vaccination and 7 days after each vaccine dose of 25 or 50 μg of MenPF1 vaccine (administered at time point 0, week 8, and week 16). Individual IgG secreting cell counts specific to PorA (A), FetA (B), and OMVs (C) in participants immunized with 25 μg (closed circles) or 50 μg (open circles) of MenPF1 are represented. The horizontal red bar represents the geometric means and 95% CI. (D) Relation between PorA and FetA-specific IgG ASC responses 7 days after the first injection. (E) Relation between PorA and OMV-specific ASC responses 7 days after the first injection.

Based on previous observations on the kinetics of B cell responses in adults following exposure to a recall antigen (17), PorA, FetA, and OMV-specific IgG antibody-secreting cell (ASC) responses were measured 7 days post each injection by *ex vivo* enzyme-linked immunosorbent spot (ELISPOT) assay without stimulation. Thus, we expected to detect mainly plasmablasts, although ELISPOT does not allow the qualitative identification like a surface marker-based assay. As anticipated, no antigen-specific ASC was detected before immunization (time point 0, Fig. 1A to C). For both PorA and FetA, the highest ASC responses were detected after the first injection (Fig. 1A and B). At this time point, 7 days post first dose, the responses to FetA in the responders ranged from 5 to 517 spot forming units (SFU)/million peripheral blood mononuclear cells (PBMC) irrespective of the dose and were of similar magnitude to the PorA-specific responses, which ranged from 7 to 450 irrespective of the dose. After the second and third doses, ASC responses were of lower magnitude for both antigens (Fig. 1A and B). The number of responders to the subdominant antigen FetA was, however, consistently lower than for PorA over the 3 time points tested (1 week after each of the 3 injections) and in both the 25 and 50 μg groups (Table 1). There was no correlation between the PorA and FetA responses at the peak of the ASC response (1-week post first injection; Fig. 1D; *P* = 0.6522). The responses to OMVs were consistently higher than for the two individual antigens (up to 1167 SFU/million cells; Fig. 1C), indicative of responses to additional antigens contained in the OMV. The kinetics of the OMV-specific ASC response was similar to the one observed with the individual antigens with the peak of response observed after the first dose. In this study, data do not suggest the dominance of the PorA-specific response in the OMV response because the PorA IgG and OMV IgG ASC did not correlate (Fig. 1E, *P* = 0.8109).

**TABLE 1:** Number (and %) of participants with a detectable IgG ASC response against PorA and FetA, 7 days after each vaccine dose

	Post 1st injection		Post 2nd injection		Post 3rd injection	
	PorA	FetA	PorA	FetA	PorA	FetA
25 μg	20/26 (77%)	7/26 (27%)	16/22 (73%)	8/22 (36%)	15/21 (71%)	3/21 (14%)
50 μg	18/25 (72%)	2/25 (8%)	16/23 (70%)	4/23 (17%)	14/23 (61%)	5/23 (22%)

### IgA-secreting ASC responses are induced by the OMV vaccine given intramuscularly.

**FIG 2:**
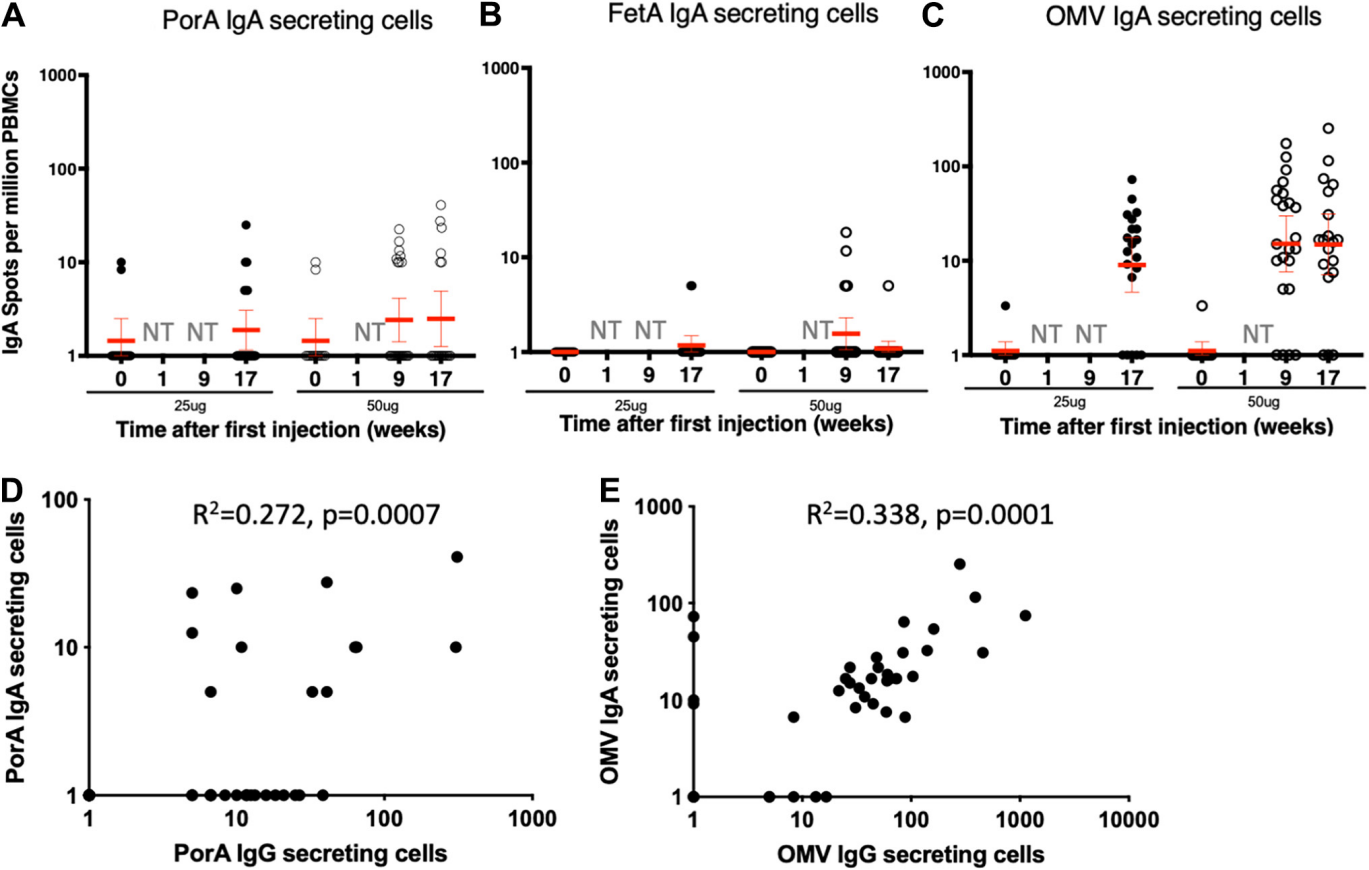
IgA-producing ASC numbers detected by *ex vivo* ELISPOT before and 7 days after the second and/or third vaccine dose of the MenPF1 vaccine. Individual IgA responses specific to PorA (A), FetA (B), and OMVs (C) are represented at the time points indicated. The horizontal red bar represents the geometric mean and 95% CI. NT = not tested. (D) Relation between PorA-specific IgG and IgA ASC responses 7 days after the third injection. (E) Relation between OMV-specific IgG and IgA ASC responses 7 days after the third injection.

Antigen-specific IgA B cell responses were induced by the MenPF1 OMV vaccine, administered intramuscularly (Fig. 2). IgA ASC responses were detected against PorA and OMVs in both groups with lower numbers of secreting cells compared with IgG secreting cells. IgA responses were low against FetA (5 to 18 SFU per million cells) and were detected in only 9% of participants in the high dose group compared with 35% against PorA, and 74% against OMVs post the second injection (Table 2). A statistically significant correlation was observed between IgG and IgA responses to PorA (*P* = 0.0007, Fig. 2D) and between IgG and IgA responses to OMVs (*P* = 0.001, Fig. 2E).

**TABLE 2:** Number (and %) of participants tested with a detectable antigen-specific IgA ASC response, 7 days after the second and third vaccine dose

	Post 2nd injection			Post 3rd injection		
	PorA	FetA	OMV	PorA	FetA	OMV
25 μg	NT	NT	NT	3/20 (15%)	0/20 (0%)	15/20 (75%)
50 μg	8/23 (35%)	2/23 (9%)	17/23 (74%)	6/19 (32%)	0/19 (0%)	16/19 (84%)

### Immunization with OMVs only marginally increases the antigen-specific IgG memory B cell responses.

**FIG 3:**
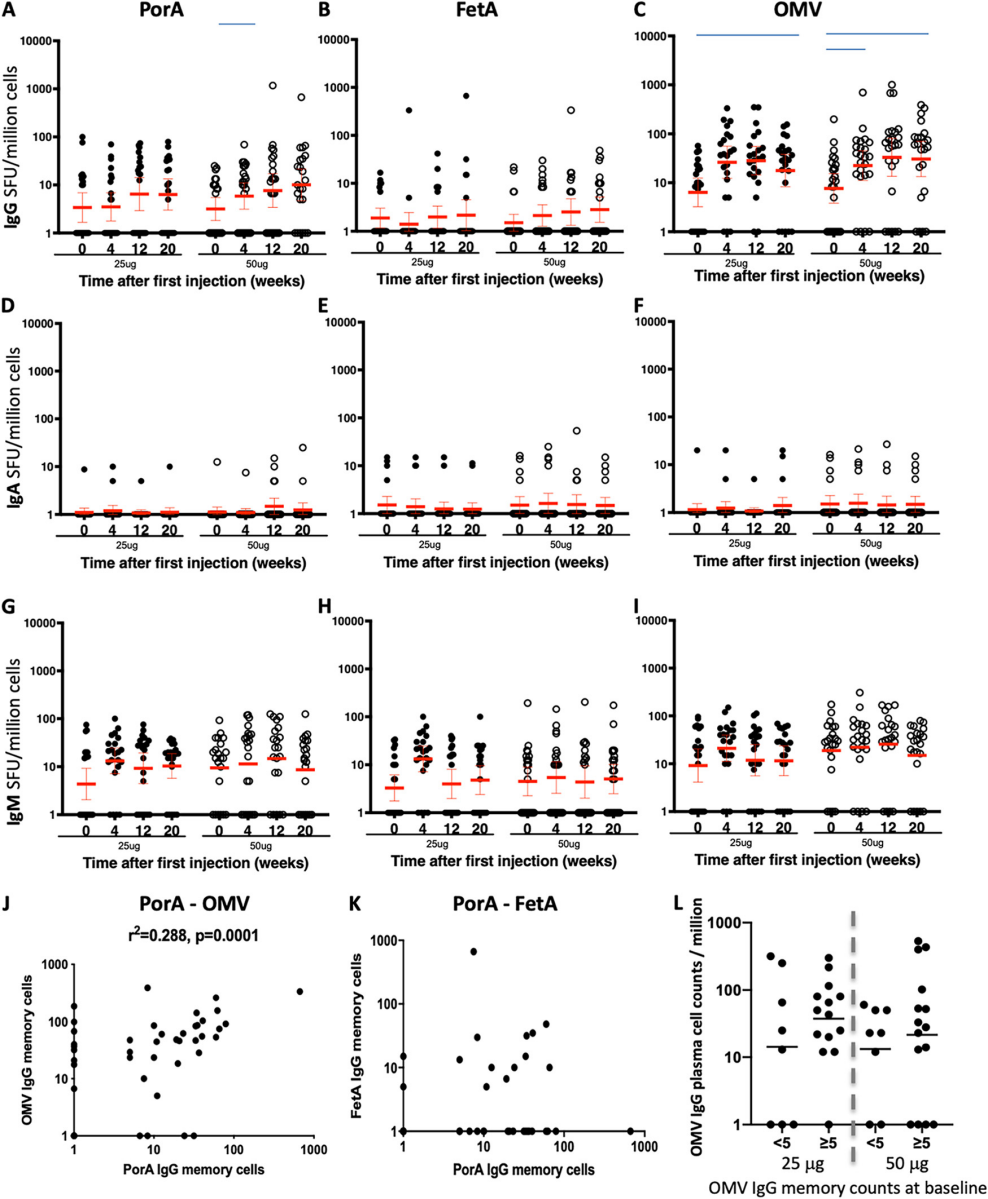
Kinetics of IgG, A, and M memory B cell numbers detected by cultured ELISPOT before and 28 days after each vaccine injection of 25 or 50 μg of MenPF1 vaccine (administered at time point 0, week 8, and week 16). Individual IgG memory B cell counts specific to PorA (A), FetA (B), and OMVs (C) are represented. The horizontal red bar represents the geometric mean and 95% CI. The blue bars denote statistical significance compared with baseline. IgA responses are represented in (D to F) and IgM responses in (G to I). (J) Relation between PorA and OMV-specific memory B cell responses after the third dose. (K) Relation between PorA and FetA-specific IgG memory B cell responses after the third dose. (L) Influence of OMV-specific IgG memory B cell response at baseline on the induction of IgG ASC responses. The individual OMV IgG ASC responses are shown for participants in each dose group according to the absence (<5) of presence (≥5) of preexisting memory B cell response to OMVs at baseline.

The number of antigen-specific IgG, IgA, and IgM memory B cells were analyzed in frozen samples before immunization and 4 weeks post each immunization using a 5-day culture to stimulate the memory B cell into antigen secreting cells. Before immunization, IgG memory B cell responses were detected in most participants (Fig. 3A to C, time 0), reflective of past exposure or carriage of meningococcus (18). IgG memory responses increased after MenPF1 injections against OMVs (*P* = 0.0250 and 0.0113 for the 25 and 50 μg doses, respectively, at week 20 versus baseline). The apparent increase of IgG memory cell responses against PorA was only statistically significant at week 4 in the 50 μg group (*P* = 0.0493, Fig. 3A to C). There were no notable increases in the number of PorA, FetA, or OMV-specific IgA or IgM memory B cells compared with baseline (Fig. 3D to I).

The PorA and OMV-specific memory IgG B cell responses after vaccination correlated (Fig. 3J, r^2^ = 0.288, *P* = 0.0001), in agreement with the idea that after OMV immunization the PorA-specific B cell response is dominant. However, IgG responses to PorA and FetA did not correlate (Fig. 3K; *P* = 0.7938). Assuming the ASC response can expand from a preexisting memory B cell pool, we assessed if the presence of a preexisting baseline memory B cell response to the antigens was associated with higher ASC responses. A trend toward a higher mean OMV IgG ASC response in participants with a preexisting memory B cell response was noted (Fig. 3L).

**FIG 4:**
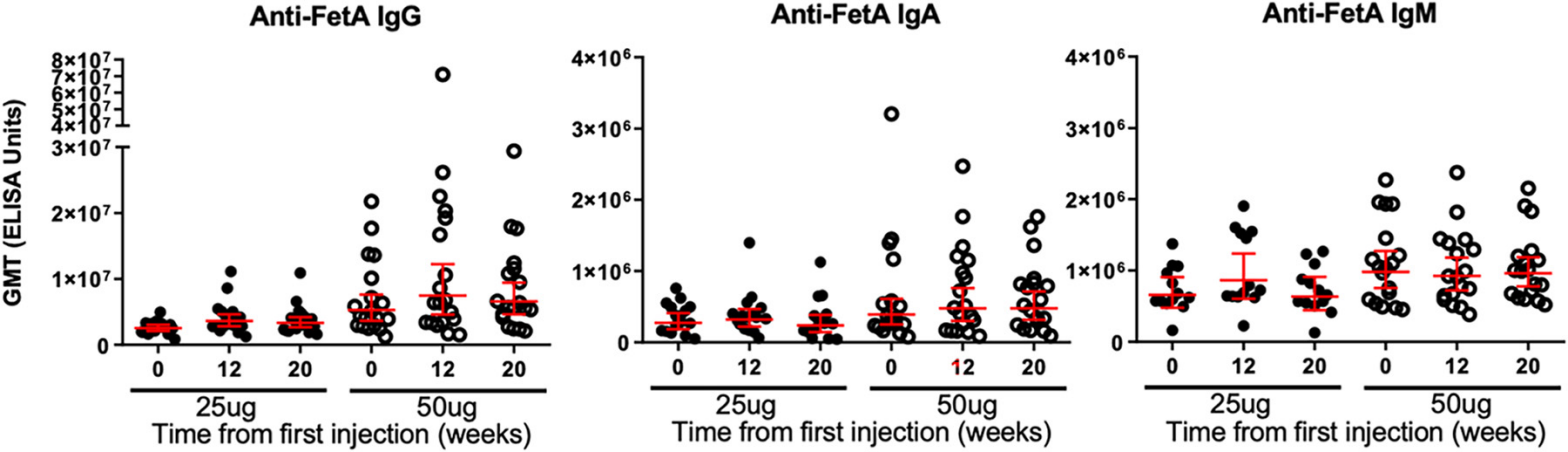
Kinetics of FetA-specific IgG, IgA, and IgM serum antibody titers before (time point 0) and a month after the second (week 12) and third vaccine doses (week 20) of 25 or 50 μg of MenPF1 vaccine (administered at time point 0, week 8, and week 16). Individual IgG (A), IgA (B), and IgM (C) are represented. The horizontal red bar represents the geometric mean of the group and 95% confidence interval.

In the context of particularly low FetA-specific ASC and memory B cell responses, we assessed whether an increase in antibody levels was detectable in serum samples (Fig. 4). The increase in serum anti-FetA antibodies was small and only observed in IgG and IgA titers in the group of participants who received the high (50 μg) dose (Fig. 4A and B) with 8/19 (42%) of the tested participants having a 2-fold increase or more in FetA-specific IgG enzyme-linked immunosorbent assay ELISA titers and 4/17 (24%) for the FetA-specific IgA response.

### The bactericidal antibody responses to the individual antigens are correlated.

To assess the contributions of PorA and FetA antibodies, individual serum bactericidal assay (SBA) and opsonophagocytic titers were measured against wild-type and four mutant meningococci. Opsonophagocytic responses were measured in the group that received the high (50 μg) dose before immunization, 4 weeks after the second injection, and 4 weeks after the third injection. The results showed that immunization with MenPF1 OMVs did not induce a significant rise in opsonophagocytic antibody responses (Fig. S1).

10.1128/mSphere.00674-21.1 FIG S1 Kinetics of opsonophagocytic responses. Kinetics of opsonophagocytic antibodies before (baseline, green) and 28 days after the second (orange) and third vaccine injection (blue) of 50 μg of MenPF1 vaccine (administered at times 0, week 8, and week 16). The opsonophagocytic fluorescence index is represented as a box and whiskers plot (min to max) against each strain tested, containing or not PorA and FetA as indicated in the x-axis. Download FIG S1, TIF file, 1.0 MB. © Crown copyright 2022. 2022 Crown https://creativecommons.org/licenses/by/4.0/ This content is distributed under the terms of the Creative Commons Attribution 4.0 International license.

**FIG 5:**
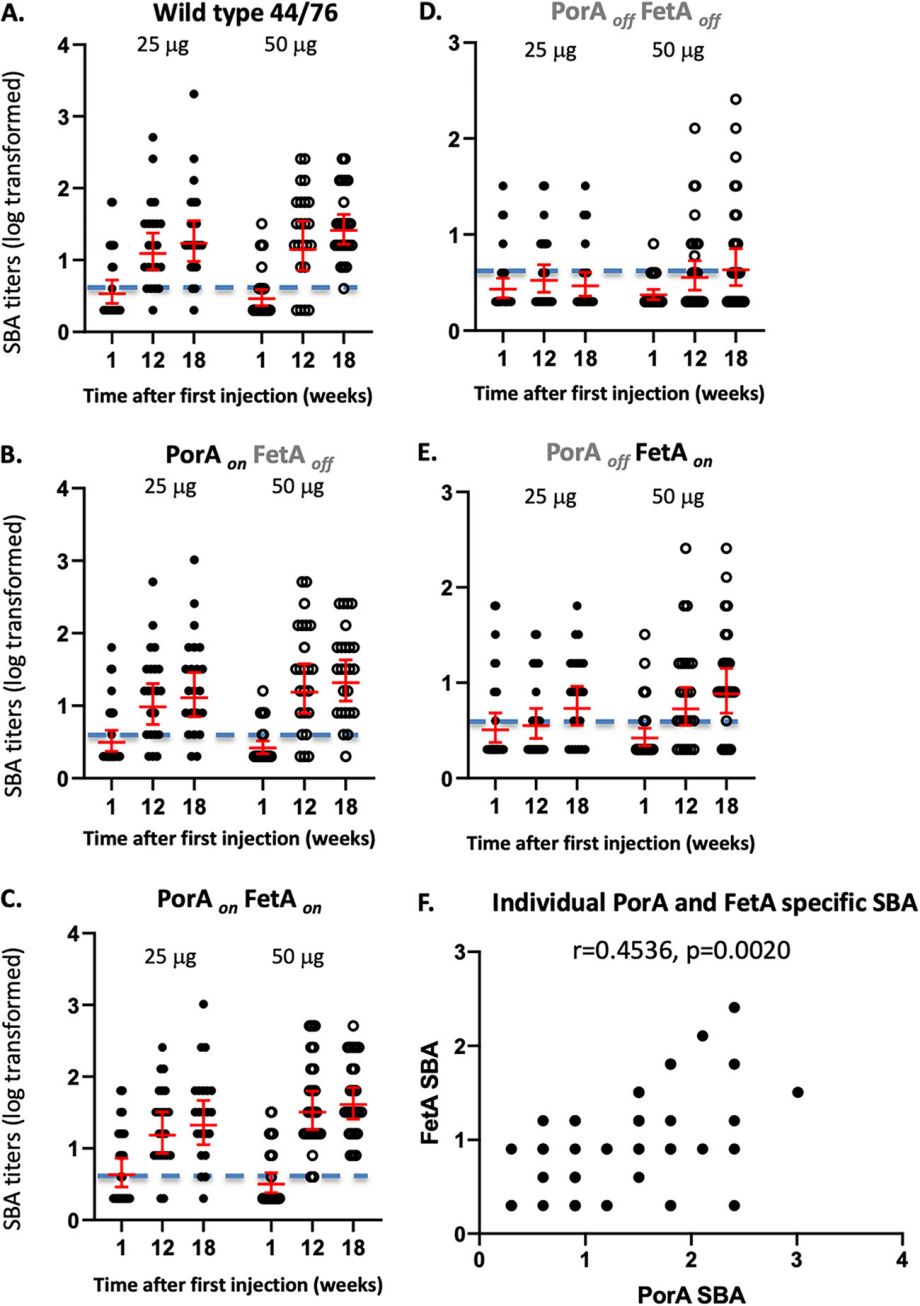
SBA titers before (time point 0) and a month post the second (week 12) and third injections (week 20) of MenPF1 vaccine (administered at time point 0, week 8, and week 16) in both dose groups. The individual SBA titers are represented against the wild-type 44/76 strain (WT; [A]) and the isogenic modified strains PorA*_on_* FetA*_off_* (B), 44/76 PorA*_on_* FetA*_on_* (C), and PorA*_off_* FetA*_off_* (D), 44/76 PorA*_off_* FetA*_on_* (E). The geometric mean per group and 95% confidence intervals are shown in red. The horizontal dotted line represents the putative cutoff for protection (titer of 1:4). (F) shows the relation between the individual PorA-specific and FetA specific titers at week 20 (both doses).

The highest increase in SBA titers was observed against meningococci that expressed PorA (wild-type, PorA_on_ FetA_on_, and PorA_on_ FetA_off_), consistent with PorA being the immunodominant protective antigen, the percentage of participants with titers > 1:4, and the geometric mean of each group at the three time points (Fig. 5 and Marsay et al. (16)). Bactericidal activity against PorA was elicited after the second OMV injection, and there was no apparent effect of a third injection or the higher dose (Fig. 5A to C). The response to FetA was lower (Fig. 5D). Three doses of OMV were required to induce a small increase in SBA response against the subdominant antigen when using the 25 μg dose while the increase was observed after two doses when using the higher 50 μg dosing schedule (Fig. 5E). This increase in SBA activity against each antigen was due to a subpopulation of the antigen-specific antibodies, and it is not surprising that there was no correlation between the SBA titers and the corresponding IgG memory B cell numbers for both antigens (data not shown). There was a relationship between the PorA-specific and the FetA-specific SBA titers at week 20 (Fig. 5F; *P* = 0.4536, Spearman *r* = 0.0020).

### Effect of preexisting antigen-specific immune response on the vaccine-induced immunogenicity.

**FIG 6:**
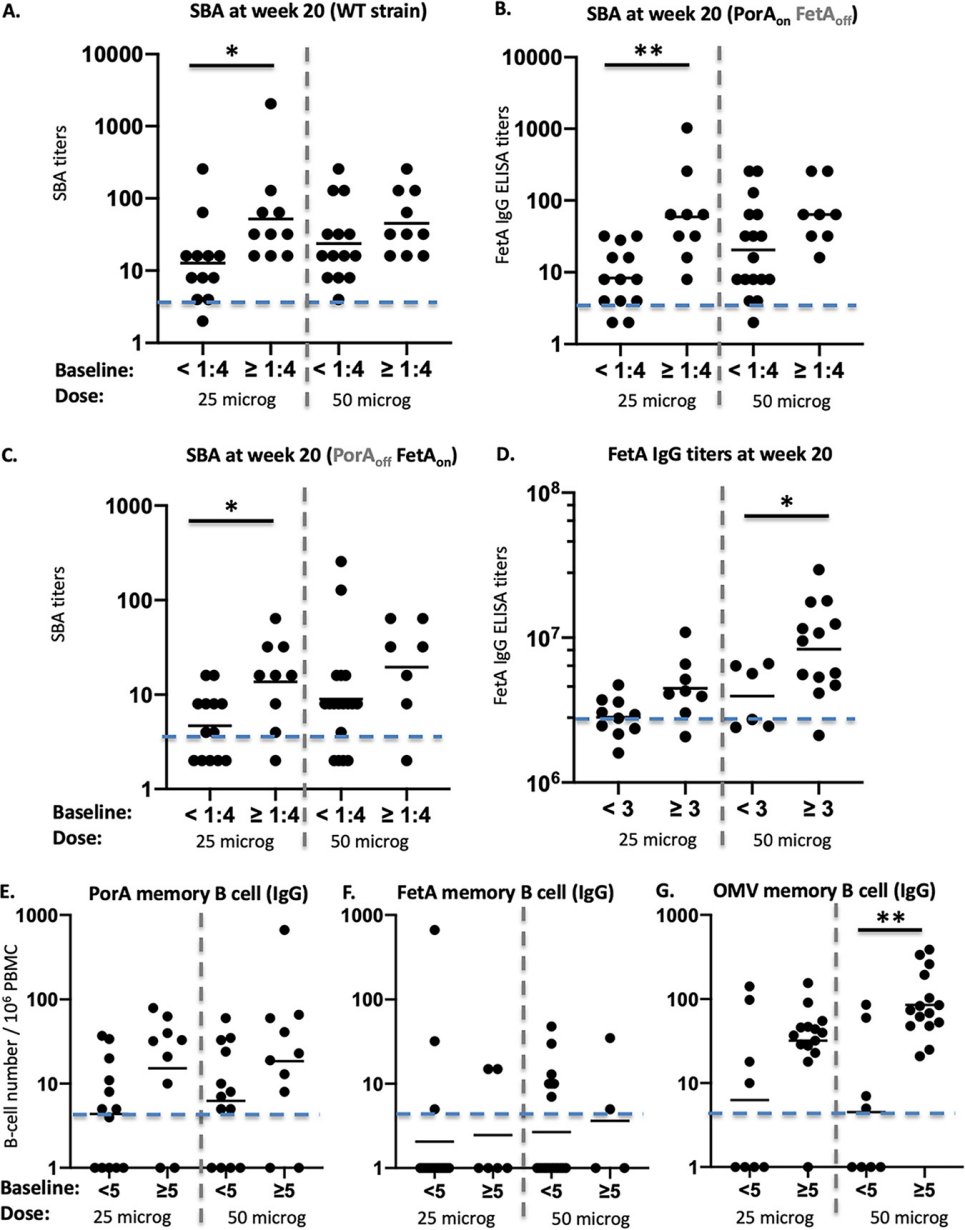
Immune responses at week 20 (4 weeks post third vaccine dose, y-axis) according to absence or presence of preexisting responses at baseline in each dose group (x-axis). Individual SBA and geometric mean titers against the wild-type strain (WT) (A) and against the PorA*_on_* FetA*_off_* (B), or 44/76 PorA*_off_* FetA*_on_* (C) are represented with participants separated in the *x*-axis according to the absence of baseline response before vaccinations (<1:4) or presence of preexisting SBA response (≥1:4) in each dose. The horizontal dotted line represents the putative cutoff for protection (titer of 1:4). Serum IgG ELISA titers and geometric mean against FetA (D). The horizontal line represents the cutoff for positivity (3 × 10^6^ ELISA titer), and participants are separated on the *x*-axis according to the absence of response at baseline ELISA titer) or preexisting response (≥3 × 10^6^ ELISA titer). The IgG memory B cell responses to PorA (E), FetA (F), and OMV (G), the horizontal dotted line represents a response >5 spots per million cells (after background subtraction) with participants separated in the *x*-axis according to the absence of baseline response before vaccinations (<5) or presence of preexisting response (≥5), in each dose. * and ** represent statistical significance as described in the text.

There was an effect of preexisting immune responses on the resulting vaccine immunogenicity (Fig. 6). Participants with protective SBA titers before vaccination (i.e., ≥1:4 at baseline) elicited higher titers at week 20 than participants with SBA responses <1:4 against the wild-type strain in the lower dose group (*P* = 0.0178). This effect was not statistically significant in the 50 μg group (Fig. 6A). The same trend was observed against the PorA_on_ FetA_off_ mutant (Fig. 6B; *P* = 0.037 in the 25 μg dose group). The response to FetA also depended on the preexisting response at baseline. SBA titers to the PorA_off_ FetA_on_ strain were higher in the participants who had preexisting FetA-specific SBA responses with significance reached in the 25 μg dose group (Fig. 6C; *P* = 0.0428). A similar trend was observed for FetA-specific serum IgG ELISA titers (Fig. 6D), although it was statistically significant at the 50 μg dose only (*P* = 0.0288).

We then assessed if the same effect of preexisting immune response influenced the IgG memory B cell responses. This was the case for the PorA and the OMV groups (Fig. 6E and G; *P* = 0.0069 for the OMV memory response in the 50 μg dose group). However, the number of participants with a detectable IgG memory B cell response to FetA was low, and no increase of response postvaccination was observed in the participants with the preexisting response (Fig. 6F).

## DISCUSSION

We performed a comprehensive analysis of the kinetics and relationships between ASC and memory B cell responses in blood against antigens included in an outer membrane vesicle vaccine, PorA (22% of the total OMV protein composition), and FetA (8% of the protein composition as determined by SDS-PAGE). This demonstrated that (i) vaccination with OMVs induces a stronger B cell response to PorA than to FetA, and (ii) three doses of OMV vaccine given intramuscularly induced an IgA-producing ASC response restricted to PorA. For the doses used, the IgG antibody and B cell responses induced by the OMV vaccine against PorA, the dominant antigen, were not dose-dependent, which was consistent with previous findings for the MeNZB OMV vaccine (19). One limitation was that this study was not powered to detect differences between the doses, or differences between readouts other than SBA responses. Nevertheless, higher OMV doses or a higher number of doses were required to induce functional immune responses (SBA) to FetA. High response to the PorA antigen was not associated with a high response to FetA with regard to quantified B cell responses, but the functional antibody titers against PorA and FetA were positively correlated.

The OMV vaccine was a poor inducer of memory B cell responses to both antigens. Preexisting memory B cell responses were detected and were only marginally boosted by the OMV vaccinations, which is in agreement with a previous study that reported that 3 doses of an OMV vaccine (VA-MENGOC-BC) were necessary to induce a detectable memory B cell response (20). In the present study, the highest SBA responses were induced in participants with a preexisting response to the antigens, suggesting that the antigen-specific B cell responses were generated from a pool of existing memory B cells. Albeit, the bactericidal antibody-producing B cells were only a fraction of the antigen-specific B cells. There was limited antibody persistence to OMV after vaccination as evidenced by the rapid waning of PorA-specific SBA after 4CMenB vaccination while the SBA specific to a recombinant protein component of this vaccine, NadA, remained elevated at the same time points (21–23). This rapid waning of immunity induced by OMV vaccines may have been due to the low OMV-induced B cell responses because a correlation of memory B cell response with antibody persistence was previously observed for a MenC vaccine in infants (24), and, in a previous study with 4CMenB, a correlation between SBA and specific memory B cell responses was observed (25). The poor memory B cell responses induced by three doses of OMV vaccine in healthy adults suggests a weak boosting capacity, providing insight into the poor persistence of antibody responses to OMV vaccines and highlighting the need for vaccines that induce better persistence and memory B cell responses.

PorA is one of the most abundant meningococcal outer membrane proteins and PorA-specific antibodies are protective against IMD (7). The antibody response to FetA can also confer bactericidal activity in humans, but, after infection, FetA-specific antibody responses are lower than responses against PorA (15, 26). Notably, FetA is present in Neisseria gonorrhea (27) with various levels of expression depending on strains and conditions. Immune responses cross-reactive between N. meningitidis and N. gonorrhea may be responsible for the modest protective efficacy of MenB OMVs reported against gonococcal disease (10). Small quantities of antigens present in OMV vaccines may boost memory protective responses induced by carriage or exposure. In this study, there was evidence of preexisting FetA-specific circulating antibodies and B cell memory response before vaccination, demonstrating that MenB carriage likely induces FetA-specific immune responses. However, while the OMV vaccine induced a bactericidal response against FetA, there was no detectable increase in the FetA-specific memory B cell response, suggesting that the FetA-specific response may be short-lived (i.e., no boost in memory B cell responses was induced) and/or that the small proportion of B cells that produce bactericidal antibodies cannot be detected within the pool of FetA-specific B cells. Moreover, our study did not indicate that other antigens induced bactericidal activity against the homologous mutants tested as evidenced by the absence of SBA response against the strain devoid of PorA and FetA.

The participants who had preexisting responses to the antigens elicited higher responses after vaccination compared with the seemingly naive participants. While this is not surprising, it is particularly interesting with regard to the effect of the MenB OMV vaccine on gonococcal disease observed in New Zealand (9). The protective effect may be dependent or limited to individuals with preexisting responses to the antigens responsible. To create more broadly protective vaccines, meningococcal OMVs can be used to induce responses to minor antigens by avoiding PorA immunodominance by (i) overexpressing the minor antigens, (ii) deleting or inactivating the PorA gene (28, 29), or (iii) performing serial immunization with OMVs containing different PorA so that only responses to the non-PorA antigens are boosted (30). Data from this study verify that the FetA protein is immunogenic as part of an OMV extracted from a FetA constitutively expressing strain.

## MATERIALS AND METHODS

### Phase I clinical trial: vaccine and participants.

The MenPF1 vaccine was manufactured from a genetically modified H44/76 N. meningitidis strain with constitutive expression of FetA (PorA variant P1.7.16 and FetA variant 3-3) as previously described (31, 32). The OMV contained 21.8% PorA and 7.7% FetA of total protein, along with other proteins derived from the deoxycholate detergent extraction of the genetically modified strain, and the final product was formulated with aluminum hydroxide (32). The study was an open-label, dose-escalation, single-site, phase I clinical trial in healthy adults as previously described (16) (MHRA reference 21584/0298/001-0001, ethical approval 12/SC/0023, clinicaltrials.gov NCT01640652 and EudraCT 2012-001046-17). Three doses of 25 μg or 50 μg MenPF-1 were given intramuscularly 8 weeks apart. Twenty-six volunteers were assigned to each dosing group by sequential allocation and invited to attend 18 visits over 20 weeks (at 0 h, 4 to 6 h, 24 h, 7, 14, and 28 d after each vaccination). Participants were healthy male and female adults aged 18 to 50 years.

### Enumeration of antibody-secreting cell (ASC) responses by ELISPOT.

Up to 120 mL of heparinized blood was withdrawn, and peripheral blood mononuclear cells (PBMCs) were separated by density gradient centrifugation as previously described (17). These were used either fresh for an *ex vivo* ELISPOT assay to detect antibody-secreting cells (ASC) or frozen in vaporized nitrogen as previously described for culture and stimulation to detect memory B cells by ELISPOT (33). For both types of ELISPOT, 96-well plates (Millipore mixed cellulose esters [MCE] membrane for IgA/IgM dual and Millipore polyvinylidene fluoride [PVDF] membrane for IgG) were coated with PorA or FetA recombinant proteins (5 μg/mL in carbonate/bicarbonate buffer), OMV, or human Ig (5 μg/mL and 10 μg/mL, respectively, diluted in phosphate buffered solution [PBS]). The cells were plated and incubated overnight at 37°C/5%CO_2_/95% humidity. For IgG ELISpot, goat anti-human IgG γ-chain-specific alkaline phosphatase conjugate (Calbiochem) was added at 1/5000 as previously described (17). For IgA and IgM ELISPOTs, antihuman IgA^Biotin^ (1/2000) and antihuman IgM^FITC^ (1/1000) diluted in PBS-4% milk was added to each well and incubated for 4 h. Plates were washed with PBS-Tween 5% and then tertiary antibodies (anti-FITC and streptavidin for the dual color IgA/IgM ELISPOT; Sigma) diluted in PBS with 4% milk were added to wells. Detection was performed with AEC (Sigma) for the IgG ELISPOT or staining liquid Vector Blue and alkaline phosphatase substrate kit (Vector Labs) for the IgA/IgM ELISPOT. Automated enumeration of antibody-secreting cell (ASC) spots was performed, optimized, and validated using an AID ELISPOT Reader ELR03 and ELISPOT software as previously described (34). Results were expressed as the number of antigen-specific spots detected per million PBMCs and subtracting the number of spots counted in the absence of antigen. A negative result was recorded as 1. A response was considered positive over 5 spots per million cells.

### Enumeration of memory B cell responses by ELISPOT.

For cell culture, PBMCs were thawed at 37°C in a water bath and added to warmed 15 mL cell recovery medium with 10 μL benzonase. Cells were washed twice and resuspended at 2 × 10^6^ in RPMI 1640 containing 5 mL Penicillin/Streptomycin and 5 mL l-Glutamate and 10% fetal bovine serum (FBS). Cells were stimulated with 1/5000 dilution Staphylococcus aureus Cowan 1 strain (SAC Pansorbin cells, Merck-Millipore, UK), 83 ng/mL Pokeweed mitogen (Sigma-Aldrich, UK) and 2.5 μg/mL CpG oligonucleotide at 37°C/5%CO_2_/95% humidity for 5 days. Cells were then washed 4 times and resuspended in medium with10% FBS. ELISPOT was performed as described above.

### Enzyme-linked immunosorbent assay (ELISA) against FetA.

High-affinity Immunolon 2HB microwell plates were coated with FetA 3-3 protein diluted in carbonate-bicarbonate solution (Sigma-Aldrich) at a concentration of 3.5 μg/mL overnight in a fridge (4 to 6°C). Wells were blocked with 1% BSA in PBS with Tween-20 (PBST) 0.05% (Sigma-Aldrich) for 2 h. Prediluted test sera, standard, and QC sera were and incubated overnight. Mouse anti-human IgG conjugate antibody diluted in 1% BSA in 0.05% PBST was added at 1:10000 at room temperature (19 to 24°C) for 2 h followed by tetramethylbenzidine for 20 min. The reaction was stopped with 1.5 M H_2_SO_4_. The optical density was measured at 450 nm. Results were expressed as standard ELISA units based on the standard curve obtained for each plate. The values of three or four sequential dilutions of each sample were analyzed to calculate the geometric mean titer (±95% confidence interval).

### Bactericidal responses by serum bactericidal assay (SBA).

Serum bactericidal titers were measured using 25% vol/vol human complement without intrinsic bactericidal activity obtained from healthy donors (clinical study OVG 2009/07, approved by the Research Ethics Committee South West 4, reference 10/H0102/23) and were published previously (16) as the percentage of participants with titers ≥1:4. Here, the individual SBA titers were reported for each dose and each time point tested. Wild-type and four mutant meningococci were used H44/76 (wild-type), FetA*_on_*PorA*_on_* (detecting responses to all antigens in the vaccine), FetA*_off_*PorA*_on_* (detecting responses to PorA and other antigens), FetA*_on_*PorA*_off_* (detecting responses to FetA and other antigens), and FetA*_off_*PorA*_off_* (detecting responses only to the other antigens).

### Opsonophagocytic activity.

The opsonophagocytic (OP) assay was performed on sera from vaccinated individuals using HL60 cells (ECACC 98070106). The H44/76-SL strain was grown to log phase, resuspended, and stained using 10 μg/mL of 2’,7’-bis-(2-carboxyethyl)-5-and-66)-carboxyfluorescein in the dark for 1 h. Bacteria were fixed with 0.2% (wt/vol) sodium azide for 48 h at 4°C. Heat-inactivated test sera diluted 1:10 in OP buffer (Hanks balanced salts solution containing 2% skimmed milk, 1.2 mM CaCl_2,_ and 1 mM MgSO_4_) was added to U-bottom 96-well microtiter plates with 10 μL of stained bacteria at 6.25 × 10^8^/mL in OP buffer and 10 μL of complement followed by incubation with shaking (250 rpm) at 37°C. A total of 50 μL of the HL60 phagocytic cells at 2.5 × 10^7^ cells/mL were added in OP buffer followed by incubation with shaking at 37°C for 7.5 min. The reaction was stopped by the addition of 80 μL of ice-cold Dulbecco phosphate buffered saline with 0.02% EDTA. Cells were washed and stained with live/dead, CD32-APC and CD35-PE, and acquired on a FACSCalibur. Gates were set against a complement-only, no-antibody control (blank). For each sample, 7,500 live HL60 cells were measured, and the percentage of cells showing fluorescence in the appropriate gate (% gated) was multiplied by the mean fluorescence of the gated population to give a fluorescence index (FI). The FI of each test was divided by the FI of the complement-only no antibody control to give a FI ratio (FIR).

### Statistics.

The sample size was calculated for the primary endpoints (safety and tolerability) for which statistical analyses were descriptively summarized and published previously (16). Immunological data were, where appropriate, log_10_ transformed before analyses. All comparisons of responses between study groups were analyzed *post hoc* using Kruskal Wallis and Dunn’s multiple-comparison tests. A repeated measure analysis of variance (ANOVA) or mixed effect analysis with Dunnett’s multiple-comparison test was used for the increase in B cell responses compared to baseline. Missing data due to laboratory processing issues were considered missing completely at random. Correlations between individual SBA titers and immunogenicity readouts were assessed on log-transformed data using Pearson’s test for B cell counts, and Spearman’s test for SBA titers. Calculations were carried out using GraphPad PRISM version 8 for Mac (GraphPad Software, San Diego, CA, www.graphpad.com).
