# Supplementary figures and images for: Human B Cell Responses to Dominant and Subdominant Antigens Induced by a Meningococcal Outer Membrane Vesicle Vaccine in a Phase I Trial
Source: mSphere. 2022 Jan 26;7(1):e00674-21. doi: 10.1128/msphere.00674-21 (PMC8791392; doi:10.1128/msphere.00674-21)

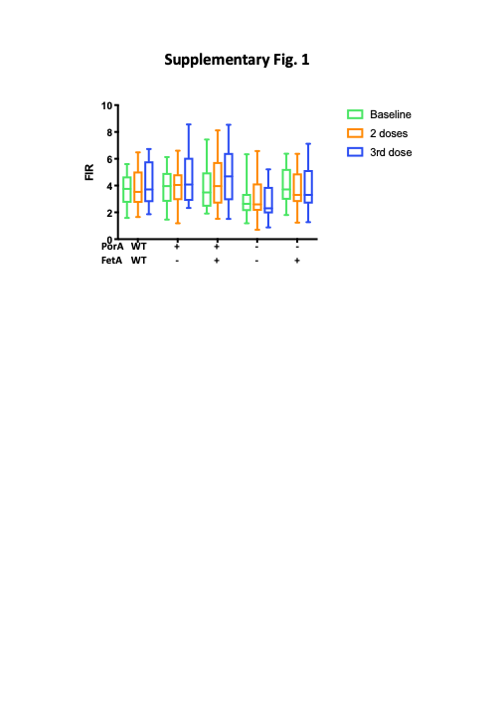

Supplement: FIG S1 [file msphere.00674-21-sf001.tif]
